# Measures, Gaps, and Mitigation Strategies in Bangladesh’s COVID-19 Response

**DOI:** 10.1007/s10393-022-01607-6

**Published:** 2022-08-10

**Authors:** Abdullah Al Sattar, Nusrat Irin, Joseph P. Belgrad, Najmul Haider, Nurun Nahar Chisty, Md. Abu Shoieb Mohsin, Mohammad Foysal, Tridip Das, Md. Helal Uddin, Rubyath Binte Hasan, Jinnat Ferdous, Mahmudul Hasan, Rashed Mahmud, Mohammed Abdus Samad, Mohammad Giasuddin, Paritosh Kumar Biswas, Dirk Udo Pfeiffer, Nitish Chandra Debnath, Guillaume Fournié, Fiona M. Tomley, Md. Ahasanul Hoque

**Affiliations:** 1 Chattogram Veterinary and Animal Sciences University, Zakir Hossain Road, Khulshi, Chattogram, 4225 Bangladesh; 2 Tufts Cummings School of Veterinary Medicine, 200 Westboro Rd, North Grafton, MA 01536 USA; 3 The Royal Veterinary College, University of London, Hawkshead Lane, North Mymms, Hatfield, Hertfordshire UK; 4 Bangladesh Livestock Research Institute, Dhaka, Bangladesh; 5 City University of Hong Kong, Hong Kong, China

**Keywords:** COVID-19, Bangladesh, Measures, Gaps, Mitigation

## Abstract

The Coronavirus Disease 2019 (COVID-19) spread rapidly from China to most other countries around the world in early 2020 killing millions of people. To prevent virus spread, world governments implemented a variety of response measures. This paper’s objectives were to discuss the country’s adopted measures to combat the virus through June 2020, identify gaps in the measures’ effectiveness, and offer possible mitigations to those gaps. The measures taken included screening device deployment across international air and land ports, flight suspensions and closures from COVID-19 affected countries, and declaration and extension of a national public holiday (equivalent to lockdowns in other countries). Identified gaps were test kit, PPE, ICU beds, and ventilator shortages, limited public awareness, and insufficient coordination and collaboration among national and international partners. Proper and timely risk mapping, preparedness, communication, coordination, and collaboration among governments and organizations, and public awareness and engagement would have provided sufficient COVID-19 mitigation in Bangladesh.

## Bangladesh’s COVID-19 Response: Measures, Gaps, and Mitigation

### Background

Severe acute respiratory syndrome coronavirus 2 (SARS CoV-2) causing COVID-19, was first identified on 7 January 2020 after Chinese authorities reported to the World Health Organization (WHO) several cases of pneumonia on 31 December 2019 of unknown etiology in Wuhan, Hubei Province, Eastern China. Subsequently, WHO declared the COVID-19 outbreak a Public Health Emergency of International Concern (PHEIC) under International Health Regulations on 30 January 2020, and then a pandemic on 11 March (WHO 2020a, b). As of June 2020, over two hundred countries and territories around the world have reported more than 10 million confirmed cases with a death toll of about 500,000 (The Johns Hopkins CHS 2020).

While COVID-19 may cause mild or asymptomatic infections, some cases can be severe, resulting in pneumonia with respiratory failure and, ultimately death (Chen et al 2020). The virus is thought to be primarily spread via direct contact and respiratory droplets (Recalcati 2020; Chan et al 2020). The disease’s incubation period ranges between 1 and 14 days, with a recommended 14-day long self-quarantine period after possible exposure to the virus (Nussbaumer-Streit et al 2020). WHO recommends basic hygiene practices such as wearing masks, regular handwashing with soap and water, and maintaining physical distance of at least 1 m (3 feet) (WHO 2020c).

Due to its high population density and weak healthcare system, Bangladesh is one of the most vulnerable countries to COVID-19. The government has implemented several measures, including traveler screening at entry ports, flight suspensions from highly affected countries, and a national quarantine. However, the daily new COVID-19 cases and death incidence have been increasing, totaling 149,258 cases and 1888 deaths reported as of June 2020 (The Johns Hopkins CHS 2020). This paper assesses Bangladesh’s COVID-19 pandemic response in the early phase of the crisis, identifies response gaps, and suggests possible solutions to those gaps. While much of the information we present in this paper is already available to the public through various media and other sources, the information is scattered without organization or analytical review. Our study is important because it extracts these data from various news outlets and other resources to form a cohesive and succinct analytical review. Furthermore, our survey results give new information regarding COVID-19.

## Materials and Methods

### Study Participants

We conducted the study at Chattogram Veterinary and Animal Sciences University (CVASU) Bangladesh. We gathered multiple perspectives on Bangladesh’s COVID-19 situation and response measures through a literature search of electronic and print media reports, relevant official documents, and interviewing pertinent participants.

Study participants were recruited through purposive sampling from the following five groups:

1. Master of Public Health Students (Bachelor of Medicine and Bachelor of Surgery, and Doctor of Veterinary Medicine)
2. Former and current Master of Veterinary Epidemiology students
3. Former and present members of the executive committee of the International Veterinary Students’ Association (IVSA) Bangladesh
4. CVASU Faculty
5. Members of the Department of Livestock Services (DLS) and the Livestock Research Organization of Bangladesh.

We chose participants who were not involved with Bangladesh’s COVID-19 crisis response so their survey answers would be unbiased, third-party opinions. Their backgrounds in veterinary or medical science provide foundational knowledge of infectious and zoonotic diseases, outbreaks, and pandemic scenarios. Some have previous experience in outbreak management with DLS, and several have past involvement with different One Health activities in Bangladesh. Given their backgrounds in veterinary medicine, public health, epidemiology, veterinary education, herd health, and outbreak management, they are well equipped to answer our two survey questions in the next section. An initial email was sent and phone calls were made to a total of 78 individuals informing them about the research and requesting their participation. We selected participants who responded to our email and phone call briefings and gave their consent to participate in the study.

### Data Collection

Our study research ethics approval (Memo No: CVASU/Dir (R&E)EC/2020/165-10). When conducting our study, we considered all available and accessible sources of news media and official documents for our literature review. Several scientific publications, national newspapers (The Daily Star, Prothom Alo, The Financial Express, The Business Standard, and The Independent), online news portals (Dhaka Tribune, Bdnews24), international news media (Al Jazeera and CNN), and official documents (World Health Organization-WHO, Ministry of Health and Family Welfare Bangladesh-MOHFW, Directorate General of Health Services- DGHS, Ministry of Public Administration and Bangladesh Medical Association-BMA) were reviewed from 20 March to June by two manuscript authors. The relevant COVID-19 pandemic data, existing gaps, etc. were extracted from these reports and entered into a spreadsheet using MS excel. Seventy-four participants participated in the study and were sent two open-ended questions, along with the objectives of the study, by email between 7–14 April 2020:

i. What response measures have Bangladesh used to manage the ongoing COVID-19 crisis?
ii. What are the potential measure gaps for managing Bangladesh’s COVID-19 crisis?

Participants were also allowed to make additional comments. We collected all responses in English from 15 to 30 April through e-mail. Some incomplete and missing responses required clarification through follow-up email communications and were re-collected shortly thereafter.

### Data Analysis

Literature review and participant response data were recorded in Microsoft Word. We conducted inductive thematic analysis identifying and interpreting participant responses. The primary researchers first familiarized themselves with the data. Two authors generated the initial codes following an inductive coding approach, finalized after several reviews. The researchers constructed potential themes after collating, combining, and comparing the emerged codes. The correspondent author subsequently reviewed the themes critically to ensure accurate code reflection. Finally, the themes were clearly defined, named, and prepared for reporting (Kiger and Varpio 2020). The results were displayed in a coding table with frequency number and percentage.

## Results and Discussion

**Figure 1:**
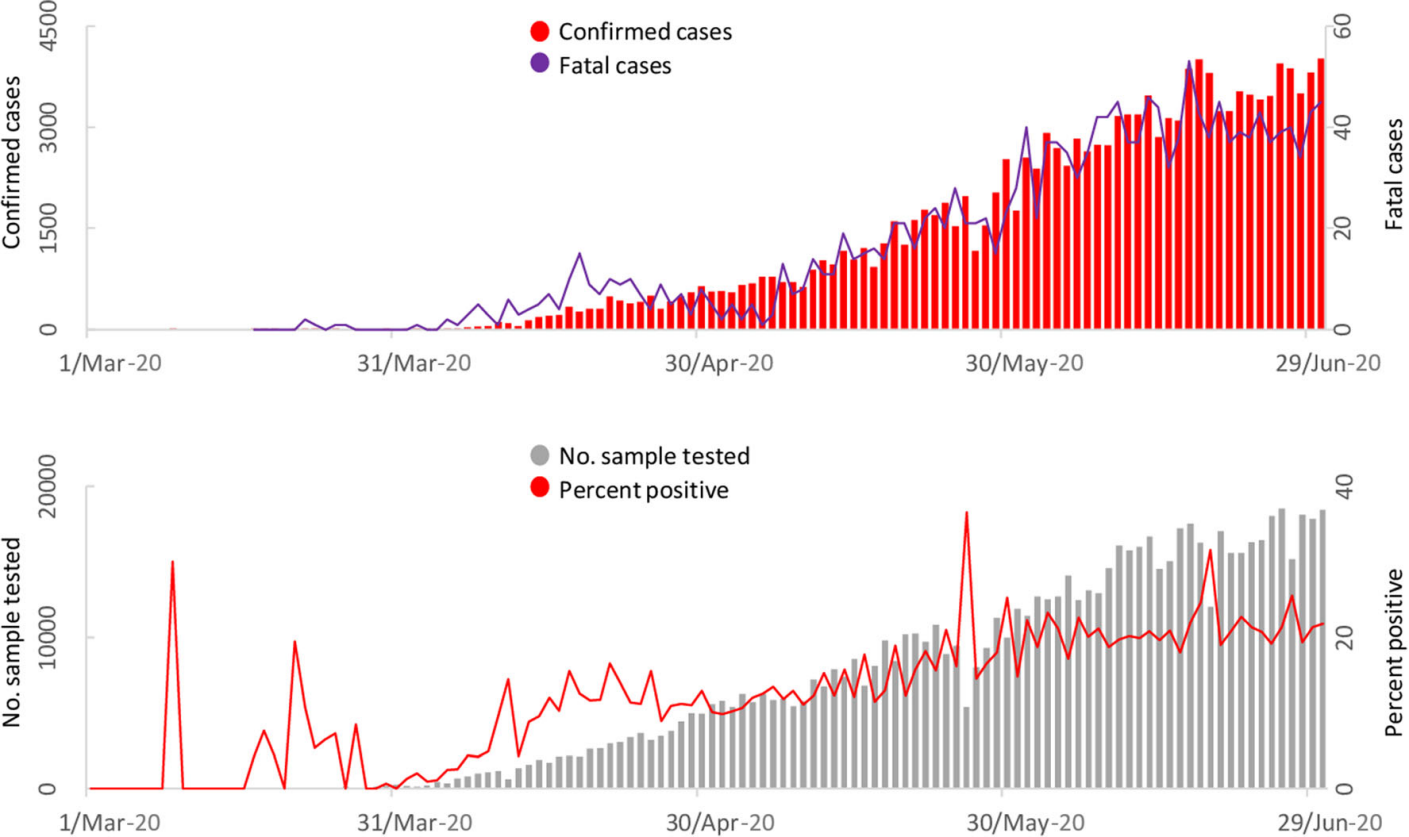
Distribution of tests, confirmed cases, and death in Bangladesh.

**Figure 2:**
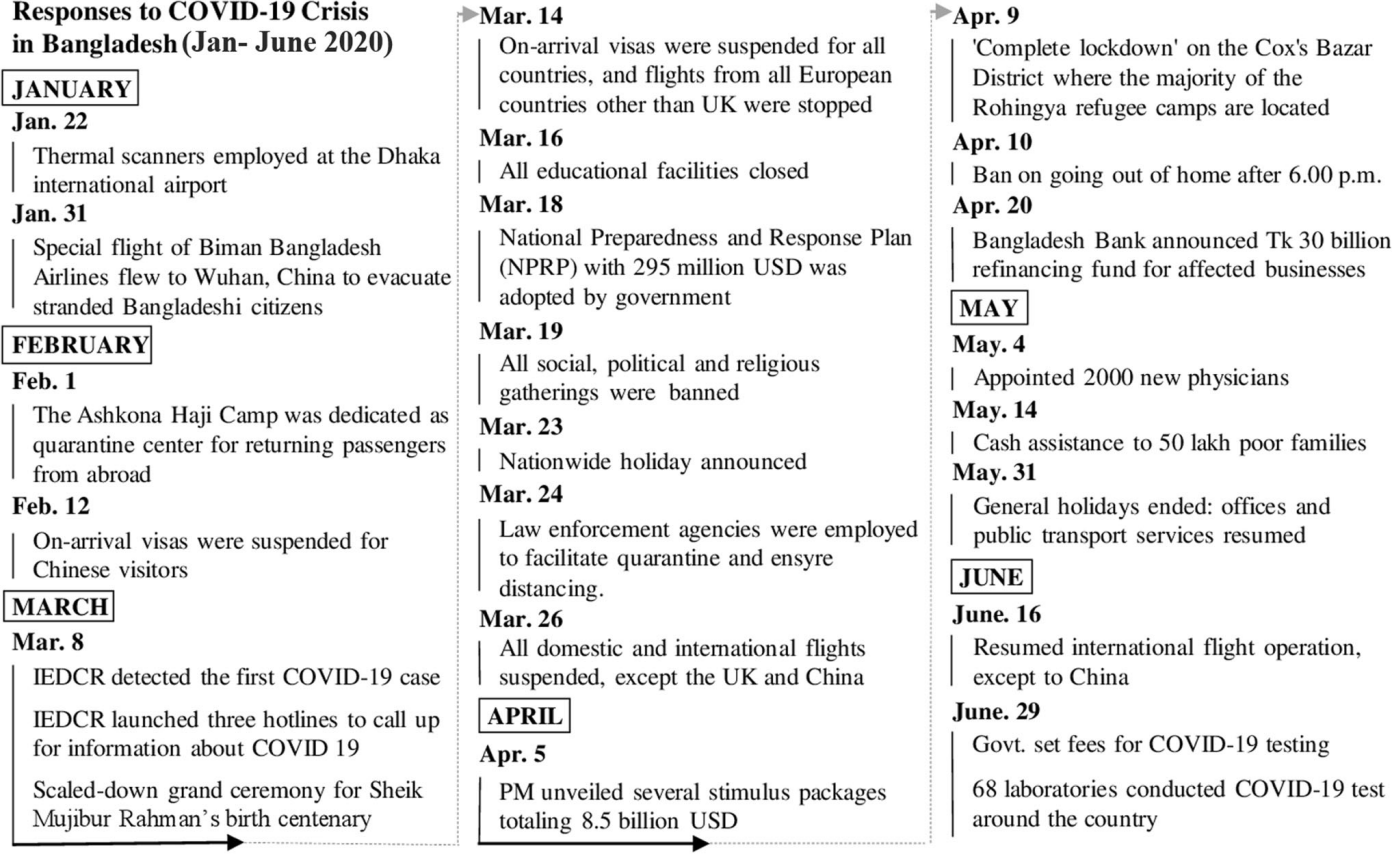
Timeline of different major responses to COVID-19 in Bangladesh.

We reviewed 51 reports/documents for the literature review. Online news portals contributed 20%, local and national newspapers 42%, and official documents 38%. Seventy-four of the 78 (95%) participants responded to the email survey (39% MPH students with MBBS backgrounds, 23% Veterinary Epidemiology MS students, 12% IVSA Bangladesh members, 12% CVASU faculty members, 12% from Livestock Research Organization of Bangladesh and 2% DLS members). Table 1 shows the generated participant response themes to our questions. Figure 1 shows the daily number of tests performed, confirmed cases, and deaths. Figure 2 shows Bangladesh’s COVID-19 major responses.

**Table 1:** Responses of Participants Against Gaps and Mitigations of COVID-19 in Bangladesh.

SL	Theme	Code	Number of responses	%
1	Communication, collaboration and coordination	Lacking in collaboration and coordination	35	47
		Lack of professionalism of Govt. personnel	17	23
		No involvement of One health, Epi, vet, NGO and others	15	20
		Incoordination of IEDCR with other organizations	2	3
2	Social distancing and movement restriction	Late and less effective lockdown	20	27
		Did not announce total lockdown/curfew around the country	4	5
		Many private organizations, bank & market still partially open	7	9
		Reopening of garments	14	19
		Not banning public and private transport	14	19
		Late suspension of international arrivals	12	16
		Late closing of borders	10	14
		People not staying at home and maintaining social distance	30	41
		The religious meeting, political meeting and election	5	7
		Rules are not followed in rural areas	2	3
3	Screening, confinement and surveillance	Inappropriate home or institutional quarantine	39	53
		Poor screening (at the airport and other ports) and surveillance	17	23
		No contact tracing	6	8
4	Diagnosis and treatment planning	Shortage of test kits, testing facilities	58	80
		Inadequate sample collection	9	12
		Not properly used facilities of other laboratories (e.g.university)	9	12
		Limited hospital, Inadequate ICU and ventilators, isolation center	56	78
		Positive patients traveled different hospitals	1	1
		Hiding real information by patients	2	3
		The reluctance of doctors to treat patients	7	9
		Other regular patients are not getting treatment	6	8
		Inadequate teleconsultation service from IEDCR	3	4
5	Ensure personal safety	Insufficient and low-quality PPEs for health workers	55	74
		Lack of enough hygiene materials	9	12
		No risk allowance for health workers	7	9
		Lack of transport and residential facilities for health workers	12	16
		No training for health workers	5	7
		No proper instructions regarding the management of hospital waste	4	5
		Inconsistency in the rational use of health care providers	2	3
6	Support, incentive and stimulus package	Dishonest and corrupted politician	15	21
		Limited support for poor	12	16
		Unpaid salary of job holders	1	1
		Announcing lock-down before providing the economic safety for mass people	1	1
		No adequate health management set up for Rohingya refugee	1	1
		Stray dogs and other animals not getting food	1	1
7	Documents, guidelines, strategies and declaration	Late response in spite of having adequate time	25	34
		No protocol to treat patients of respiratory disease other than COVID-19	3	4
		No systemic control strategy	5	7
		Less budget in health and research sector	3	4
		Poor medical infrastructure	2	3
		Old epidemic law	1	1
		No tendency of decentralization	1	1
		Failure to activate the Incident Management System (IMS) and Health Emergency Operations Center (HEOC) in spite of having adequate time	1	1
		No research (risk factor, vaccine, drug) in BD	3	4
8	Public awareness	Lack of public awareness	23	31
		Not involving religious leaders and places for raising awareness	1	1
		Blaming doctors by govt and people	1	1
		Mass media fail to create awareness in time	4	5
9	Press conference/health bulletin	Hiding information by govt and IEDCR	10	14
10	Dealing rumors	Inadequate response against superstitions and rumors	7	10

### Communication, Collaboration, and Coordination

The government of Bangladesh (GoB) has partnered with multiple national and international entities responding to COVID-19 such as the WHO, UN, World Bank, and South Asian Association for Regional Cooperation (SAARC). The government has committed USD 1.5 million to SAARC’s COVID-19 emergency fund. On 19 April, in response to COVID-19, the Health Service Department and the Ministry of Health and Family Welfare (MOHFW) formed a joint 17-member National Technical Advisory Committee whose responsibility was to advise the government on improving medical services and enhancing health professional skills (WHO 2020d). Local administrations, law enforcement agencies, and armed forces were deployed to facilitate quarantine measures and ensure social distancing (The Independent 2020a). Respondents (58%) said this measure was effective.

Some respondents (47%) said a lack of coordination and collaboration between the government, its agencies, the private sector, and UN agencies hindered the GoB COVID-19 response. Other respondents (23%) said some official and influential statements downplaying the virus’ severity were unprofessional and potentially dangerous.

Several (20%) respondents expressed that collaboration among animal, human, and environmental sectors in a One Health would facilitate problem identification and solutions.

### Social Distancing and Movement Restriction

In response to COVID-19, Bangladesh implemented an on-arrival visa suspension for visitors from China on 12 February and all European countries except the UK on 14 March. International flights were suspended until 15 June 2020, except for special flights repatriating Bangladeshi nationals abroad and repatriating foreign nationals in Bangladesh (WHO 2020e). Many political, social, cultural, and religious events were banned starting in March. State events including Independence Day and Bengali New Year celebrations were canceled, and the grand ceremony for the birth centenary of the Father of the Nation Bangabandhu Sheikh Mujibur Rahman was minimized (Al Jazeera 2020). All schools and educational facilities were closed on 16 March until further notice (WHO 2020f). On 23 March, a ten-day nationwide holiday was declared with all but essential services closed. The holiday was later extended until 30 May 2020 (WHO 2020g). Religious gatherings were restricted to limited devotees in the mosque during Ramadan (WHO 2020h). Most participants (53%) thought the public holiday was effective. However, other participants (19%) said public transportation available during the holiday potentially spread the virus further. Banning mass gatherings (33%), flight suspensions (33%), and other movement restrictions (34%) were also thought to be effective measures.

Some respondents (16%) felt the government was delayed in its flight suspensions likely due to the repatriation process. The government took many steps to close down businesses and institutions, however, it could have done a better job of closing transportation. Several study participants (19%) and the literature review (Kabir et al. 2020) identified certain inconsistencies in various industries, including the re-opening of garment factories (26 April) despite public holiday extensions and employee salary withholds.

Both the literature review (Dhaka Tribune 2020a) and the survey results (41%) recognized that many people, regardless of socio-economic status, outright refused to maintain social distance or remain in self-quarantine, often going out into public without safety precautions despite guidelines on social distancing in effect. Uncontrolled social and religious events likely contributed to the virus spread (CNN 2020). Due to Bangladesh’s massive population and low economic status and education, social distance enforcement, as recommended by the WHO, is impractical. The Bangladesh government should develop a standard operating procedure in coordination with health and epidemiological experts to prepare for potential future outbreaks.

### Screening, Confinement, and Surveillance

Hazrat Shahjalal international airport, Dhaka started traveler health screenings with thermal scanners on 22 January 2020 for COVID-19 symptoms (such as high body temperature, running nose, cough, etc.) and epidemiological linking. This arrangement was extended to other airports, seaports, land ports of entry, and the cantonment rail system where a total of 736,155 people were screened as of June (DGHS 2020a). Bangladesh imposed an obligatory 14-day quarantine period overseas. The Ashkona Haji Camp, Dhaka was dedicated as a quarantine center where Bangladeshi returnees from China and Italy were quarantined in February and March (Dhaka Tribune 2020b). As of June, around 629 quarantine centers were earmarked across the country serving more than 30,000 people (DGHS 2020a).

The local administration locked down COVID-19 hotspots with 400 Upazilas from 50 districts under complete lockdown until April. Public transportation was suspended, citizens were asked to remain home and all businesses except grocery and drug stores were instructed to close (WHO 2020f). The respondents (45%) said the government's implementation of home quarantine and isolation facilities were effective. Respondents (36%) also deemed screening facilities and locational lockdowns effective.

Fifty-three percent of the participants believed that inappropriate quarantine strategies for overseas returnees were one of the major reasons for the virus’ spread. Returnees’ refusal to remain quarantined hampered the government’s effort to control the virus (The Financial Express 2020a). Fear and social stigma hindered early health investigations and case detection (Pokharel et al 2020). Many of the respondents noted inappropriate screening and surveillance (32%) and contact tracing (8%). Limited manpower and non-functional machinery hindered screening procedures at the point of entry (PoE), and scarce databases on health systems prevented effective surveillance and monitoring systems production (Huq and Biswas 2020).

### Diagnosis and Treatment Planning

The Institute of Epidemiology, Disease Control and Research (IEDCR) remained the centralized sample collection and testing facility after the first COVID-19 case was detected on 8 March 2020. The government eventually approved 68 other laboratories (30 June 2020), including several universities and private hospitals across the country, to conduct COVID-19 testing (DGHS 2020a). The government has imported 100,000 testing kits from China to facilitate diagnostic procedures. The MOHFW arranged many COVID-19 dedicated hospitals (twenty in Dhaka and eighty outside the capital) with separate units and improved oxygen supply and ventilator facilities (MOHFW 2020). Several special field hospitals have been established with help from political leaders, influential personnel, and public and private organizations (The Daily Star 2020a; b). As of April, the government has set up 192 ICU beds for treating COVID-19 patients, 79 of which are in Dhaka (Anik and Hasan 2020).

Patients and doctors can be connected through a toll-free hotline with telemedicine services through the IEDCR. In May 2020, the government newly recruited 2000 doctors and 5000 nurses appointed at dedicated COVID-19 hospitals across the country (Ministry of Public Administration 2020). The participants said that testing facilities (45%), dedicated hospitals (42%), and the coronavirus helpline (17%) were effective measures.

Participants identified the lack of testing kits (80%) and insufficient hospitals with proper treatment facilities (78%) as critical response drawbacks. The testing rate in the first two months of the pandemic was negligible with less than 200 tests per million people. Although testing has increased, it was lower than in neighboring countries in South Asia (4410 per million as of June) (WHO 2020i). The respondents indicated shortages of physicians, essential medical facilities including ventilators, ICU beds, and proper medical waste disposal systems. With only one physician for every 1581 people, one hospital bed for every 1196 people, and insufficient medical equipment (192 ICU beds and 1769 ventilators), the government struggled to deal with the pandemic (Khatun and Saadat 2020; Anik and Hasan 2020; Islam et al 2020). False COVID-19 test results, false treatment, and testing fees also reduced testing frequency (The Business Standard 2020a; b).

### Ensure Personal Safety and Training Program

The government set up appropriate training programs for health care professionals and ensured their safety by providing them with the required (2,362,814 distributed and 165,431 stored through June 2020) (MOHFW 2020). The government has imported PPE from abroad, and several local entrepreneurs have stepped-up efforts to produce PPE locally. Various universities, organizations, and ready-made garments (RMG) volunteered to produce and distribute sanitizer, masks, and PPE. The DGHS facilitates video training for hospital staff to ensure personal safety in caring for and managing COVID-19 patients with around 4000 physicians and 1500 nurses receiving training by April(WHO 2020d). A medical team from China, comprising 10 respiratory specialist doctors experienced in confronting the COVID-19 pandemic, visited several hospitals in Bangladesh and shared experiences of treating and nursing mild and critical cases with frontline doctors (The Business Standard 2020c). The DGHS conducted online training sessions on the burial of suspected or confirmed COVID-19 cases. Approximately 5500 people have been trained so far (WHO 2020d).

Over half the respondents (53%) acknowledged that PPE production and training were effective, however, 75% of respondents said there was still a deficiency in PPE and training for healthcare providers. As of June 2020, more than 50 physicians have died, and more than 5000 health care professionals have been infected with COVID-19 in Bangladesh (Bangladesh Medical Association 2020). There were allegations of government corruption in distributing masks, personal protective equipment (PPE), and other materials. Those responsible for this corruption were removed with legal action taken against the supplier company (The Financial Express 2020b).

### Support, Incentive, and Stimulus Package

On 5 April, the Prime Minister unveiled several stimulus packages totaling BDT 72,750 core (around 8.5 billion USD and about 2.5% of GDP) to counter COVID-19’s effects on the country’s economy (WHO 2020j). Since mid-May, the government has been dispersing cash assistance through major mobile financial service providers (The Business Standard 2020d). It was decided to allocate Tk 1.0 billion in the next fiscal year for the ‘Rural Social Services Programme’ to stimulate the rural economy in the aftermath of the COVID-19 pandemic (The Financial Express 2020c). The government announced special insurance and stimulus packages for frontline workers including health workers and other emergency service providers (The Financial Express 2020d). These incentives and insurance were largely (60%) noted as effective.

Some respondents (21%) and literature acknowledged several instances of corruption that prevented the proper distribution of relief and cash support (The Daily Star 2020c). Therefore, an enriched database was developed, and smart cards were issued to remove duplications, eliminate ghost lists, and reduce corruption. Many officials involved with corruption have been terminated as a result. The government should take the measures recommended by the world bank such as providing safety nets; securing access to food, medical supplies, and necessities for citizens; enacting debt relief measures for trades; and adopting expansionary fiscal policies to keep credit flowing in the economy afterward (World Bank 2020).

### Documents, Guidelines, Strategies, and Declaration

On 18 March 2020, GoB adopted the National Preparedness and Response Plan (NPRP) for COVID-19 with a total cost of USD 29,550,000 million. Subsequently, a multi-sectoral Country Preparedness and Response Plan (CPRP) was developed through the collaboration of the UN to support the NPRP (WHO 2020f). On 8 April, the Bangladesh Multi-sectoral Action Plan for COVID-19 was adopted to support MOHFW through the health Emergency Response, the Humanitarian response, and post-pandemic socio-economic recovery assistance (WHO 2020k). Aligned with the latest evidence and WHO guidelines the DGHS and MOHFW prepared 34 guidelines (as of June) on Standard Operating Procedures (SOP) for hospital management, homecare guidelines, Infection Prevention and Control (IPC), rational use of PPE, waste management, and burial/final disposal of the dead body (WHO 2020j). The daily updates (cases, deaths, tests, and other demographics) and other precautionary measures were briefed (till mid of August) every day by the Ministry of Health through national television, radio, and social media.

Although, the participants (12%) acknowledged the NPRP’s strategies and guidelines for COVID-19, one-third of them (34%) believed that it was delayed and might remain on paper and ineffective mostly due to poor infrastructure, insufficient resources, and technical limitations. Failure to activate the Incident Management System (IMS) and Health Emergency Operations Center (HEOC) in timely, inadequate data availability, and poor investment in research and development were also mentioned as gaps in government responses. Despite nearly three months’ notice since the first COVID-19 case in China, insufficient, and at times inappropriate, actions were taken in response to the outbreak. Some (8%) respondents highlighted the government’s inadequate investment in scientific research narrowing Bangladeshi scientists to theoretical researches on COVID-19 rather than intensive laboratory experimentations. Sociological research might be helpful to identify the social realities and socio-economic implications of taken measures and policies. Epidemiological and field research would support identifying risk factors, transmission processed, and disease variations.

### Public Awareness

The government has been educating citizens on the importance of social distancing and proper personal hygiene using various media outlets. WHO’s instructions and guidelines were disseminated to the public continuously through mass media and social media. Local law enforcement agencies have given notice and relayed recommendations (DGHS 2020b). Most of the respondents (56%) identified the aforementioned activities as an efficient approach to educating the public but 31% of respondents thought that these awareness activities were not properly executed. Initially, inconsistent government statements COVID-19’s created mass confusion (Bdnews24 2020). A large portion of the population is not concerned about the pandemic and has no safety precautions planned. Hand sanitizer and face mask shortages have slowed public response, and many people were ignorant of proper social distancing (Islam et al 2020).

Low literacy rate, poverty, and low socioeconomic status all contribute to decreased public awareness. Political and social laders should partner with media outlets to emphasize the WHO guidelines to support educating the public. Social workers, volunteers, human doctors, and veterinarians can also play an active role in raising public awareness during the pandemic.

### Press Conference/Health Bulletin

The Ministry of Health and IEDCR publishes updates on the number of samples tested, infected patients, death toll, recovered patients, most infected districts, number of home quarantined individuals, supply and availability of PPEs, and the overall COVID-19 situation through various mass media sources such as television, radio, and social media. The health bulletin on COVID-19 is televised and published regularly. Later, the MOHFW lead the health bulletin without receiving any questions from any news reported. While informational, more statistics on the daily and total cases and their temporal and spatial dynamics would be more helpful. Several respondents (14%) feel the bulletin could do a better job of maintaining transparency between the government and the people as the total number of cases and the death toll have not always been provided. It was proven from the previous pandemic that timeliness and transparency in sharing real information might make the public more responsible rather than creating panic (Tan 2006). We can see that absence of complete information prevents the public from understanding the severity of the situation leading to people ignoring recommended guidelines.

### Dealing with Rumors

The superstitious and superabundance of information relating to COVID-19 on news and social media has emerged as a major problem worldwide making it challenging to differentiate fact from fiction. Like many other countries, rumors spread over the contagiousness of the diseases across the country such as the view that COVID-19 is just the seasonal flu, not so fatal, or won’t do much harm in the tropical environment of Bangladesh. In April, fake news about the probable death of 2 million Bangladeshi due to COVID-19 created mass panic (Daily US Times 2020). Fictional news about different cures and medicine spread everywhere. Social media are the source of rumors, misinformation, and various superstitions. Rumors, especially those on Facebook, got media attention and were addressed by the Ministry of Information. The government strictly took harsh actions against false claims, rumors, and other misinformation (Alo 2020). Local leaders, mosque Imams, and other religious leaders were requested to share hygiene and physical distancing guidelines. Several participants (10%) criticized the government's inadequate response to the rumors at the outset, but more of them (30%) described the steps taken by the government as appropriate, timely, and effective. However, social scientists and anthropologists were not involved in the national committee. Consulting these groups in a One Health manner could have made a difference in addressing behavioral changes, family planning, immunization, and sanitization. Social scientists and animal health specialists working together would facilitate proper public education.

## Study Limitations

Some potential weaknesses of this study are some self-selection biases of the survey participants. Since we only surveyed those who responded to our requests to survey, the participants were self-selected. Another potential weakness is the possibility of news biases. Much of our data was collected from news media sources. Media funding sources may encourage news outlets to spin stories in a certain perspective that can bias the information being delivered. Another limitation was that we took responses from those in the veterinary and medical field. The COVID-19 pandemic is a complex and multi-dimensional issue in terms of its response mechanism, community response strategy, public health measures, and response from the health system. Responses from social scientists, social workers, administrators, and the other forcing entities closely related to the COVID-19 response would add a more well-rounded study offering different viewpoints.

## Conclusion

COVID-19 has a devastating effect on Bangladesh. The government has adopted many measures to reduce the spread of COVID-19 across the country. However, there are some important areas where the government should increase attention such as obtaining more testing equipment and PPE, increasing ICU capabilities, ventilator facilities, and social awareness. The engagement of locally elected public representatives and field health workers in conjunction with traditional media sources would assist in generating public awareness. As discussed in the results and discussion section, collaboration among human, animal, environmental, and social health in a One Health Approach will help to strengthen the country’s COVID-19 response. Therefore, GoB should apply an effective implementation of this holistic approach.
